# Prevalence of the Oral Corticosteroid Exposure and Excessive Use in Patients with Inflammatory Bowel Disease: Data from Four French Referral Centers of the International DICE Study

**DOI:** 10.3390/jcm13092652

**Published:** 2024-04-30

**Authors:** Stéphane Nancey, Xavier Hébuterne, Cyrielle Gilletta, Evguenia Hacques, Xavier Roblin

**Affiliations:** 1 Hépato-Gastro-Entérologie, CHU de Lyon Sud, 165 Chemin du Grand Revoyet, 69495 Pierre-Bénite, France; stephane.nancey@chu-lyon.fr; 2 Faculté de Médecine, Université Claude Bernard Lyon 1, 43 Boulevard du 11 Novembre 1918, 69622 Villeurbanne cedex, France; 3 Gastro-Entérologie et Nutrition Clinique, CHU Hôpital Archet 2, 151 Route St Antoine, 06200 Nice, France; hebuterne.x@chu-nice.fr; 4 Faculté de Médecine, Université Côte d’Azur, Avenue Valrose, 06000 Nice, France; 5 Gastroentérologie et Pancréatologie, CHU de Toulouse, Hôpital de Rangueil, 1 Avenue Pr Jean Poulhes, 31059 Toulouse, France; gilletta.c@chu-toulouse.fr; 6 Affaires Médicales, AbbVie, 10 rue d’Arcueil, 94528 Rungis cedex, France; 7 Gastro-Entérologie et Hépatologie Maladies Inflammatoires, CHU de Saint Etienne, Hôpital Bellevue, 25 Boulevard Pasteur, 42100 Saint Etienne, France; xavier.roblin@chu-st-etienne.fr; 8 Faculté de Médecine, Université Jean Monnet, 10 Rue Tréfilerie, 42100 Saint Etienne, France

**Keywords:** corticosteroids, Crohn’s disease, inflammatory bowel disease, ulcerative colitis

## Abstract

**Background**: Corticosteroids used to induce a response in Crohn’s disease (CD) and ulcerative colitis (UC) may cause adverse reactions. The DICE study aimed to quantify and investigate factors associated with their use. **Methods**: This cross-sectional, non-interventional study conducted in seven countries allowed us to collect data on oral corticosteroid exposure and excessive use (cf. British Society of Gastroenterology) over the past 12 months in adult patients with CD or UC for more than a year. The factors associated with these practices were investigated using marginal logistic models. We present the results from the four participating French expert centers. **Results**: Corticosteroid exposure over the past 12 months was observed in 20.1% of 324 CD patients and 30.2% of 205 UC patients. Excessive use was reported in 13.3% and 17.1% of patients, respectively. Corticosteroid exposure and excessive use were less frequently observed in CD than in UC (OR: 0.56, *p* < 0.0001, and 0.69, *p* = 0.0042). A disease activity assessment at patient’s last visit was the main factor (*p* < 0.01) associated with the risk of corticosteroid exposure and excessive use in CD (OR: 3.41 and 3.44) and UC (OR: 7.29 and 6.90). **Conclusions**: Corticosteroid exposure and excessive use continue to be frequently observed in CD and UC in France.

## 1. Introduction

Inflammatory bowel disease (IBD) is characterized by inflammation of the bowel wall due to the dysregulation of the immune system. It is characterized by alternating flares and remission and can lead to complications (fistulae, abscesses, stenosis, and gastrointestinal perforation), with an increased risk of colorectal cancer favored by chronic bowel inflammation [1,2]. In 2019, 273,100 individuals were affected by IBD in France, 53% of whom were females [3]. In 2014, the incidence rate of Crohn’s disease (CD) in Northwestern France was assessed at 7.6/100,000 inhabitants, with a significant increase since 1988, whereas the incidence rate for ulcerative colitis (UC) remained stable at 4.4/100,000 [4]. A clear increase in the incidence of both types of IBD was observed in adolescents during this period.

The short-term therapeutic objectives for both diseases include symptom relief and the induction of remission [5,6]. In this context and despite the therapeutic progress achieved with the development and growing use of biotherapies, corticosteroids (CSs) continue to be a major treatment for moderate-to-severe IBD flares. However, although the efficacy of CSs is recognized in terms of inducing the remission of the disease, remission on CSs is not long lasting [7]. Furthermore, CSs cause numerous adverse effects, particularly in the case of high doses and/or prolonged use (insomnia, weight gain, hypertension, hyperglycemia, cataract, glaucoma, osteoporosis, Cushing’s syndrome, venous thromboembolism, mood disorders, the increased risk of opportunistic infections, etc.) [8,9], and even increased mortality in comparison with tumor necrosis factor alpha inhibitors (TNF-alpha inhibitors) [10]. The benefit/risk ratio of CSs in IBD thus remains a topical issue in current medical practice, as is the case for other chronic inflammatory diseases, such as rheumatoid arthritis or lupus, which has led to recommendations for the use of CSs in these diseases [11,12].

International and French recommendations have been published, in this context, to limit oral CS use, treatment duration, and doses in CD or UC patients [13,14,15,16,17]. Excessive CS use, which is most often linked to resistance/dependence to treatment, has been defined in IBD by the British Society of Gastroenterology [13] according to at least one of the following four criteria: (a) >1 course of CSs within the last 12 months, (b) CS use for ≥3 months within the last 12 months, (c) the inability to reduce CSs below the equivalent of 10 mg/day prednisolone (or 3 mg/day budesonide) within 3 months of starting CSs without recurrent active disease, and/or d) relapse within 3 months of CS discontinuation. 

In this context, the objective of this study was to describe oral CS exposure and excessive use in IBD patients and to search for the associated factors. We present the results collected in France below.

## 2. Materials and Methods

### 2.1. Study Design

DICE INCIDENCE CAPTURE (Determinants, Incidence, and consequences of Corticosteroid Excess in IBD) is an international, cross-sectional, non-interventional study conducted in seven countries, including France. The study was conducted in France between September and November 2021. The participating hospitals completed an online questionnaire for all adult patients (age ≥ 18 years) diagnosed with IBD for more than a year. The study excluded patients with suspected but unconfirmed IBD, UC after colectomy with no evidence of CD, taking part in a clinical trial evaluating a treatment, or with concomitant conditions (solid organ transplantation, adrenal insufficiency/Addison’s disease/hypopituitarism, autoimmune hepatitis, polymyalgia rheumatica, uveitis, systemic lupus erythematosus, rheumatoid arthritis, giant cell disease, vasculitis, hemolytic anemia). As data collection from patients included in the study was completely anonymous (no direct or indirect identifying data), prior authorization from an ethics committee and patient consent were not required.

### 2.2. Data Collected

The following data were collected on the patients included in the study: type of IBD (CD, UC, or unclassified colitis); previous and current IBD treatments; disease activity at the last patient assessment; oral CS exposure within the last 12 months with, in this case, any prescribed osteoprotective medications; and data used to assess possible excessive use (number of courses of CS, longer duration of use, possible dose reduction below the equivalent of 10 mg/day prednisolone or below 3 mg/day budesonide within 3 months of starting CSs without recurrent active disease, and any relapse within 3 months of CS discontinuation).

### 2.3. Statistical Methods

The previous and current treatments for CD and UC were described, along with the number of patients exposed to CSs and having used CSs excessively within the last 12 months, stratified by type of IBD, with the associated odds ratio (OR) and 95% confidence intervals (CI) (marginal logistic regression taking into account the correlation between patients managed at a given center). Analysis of the factors associated with CS exposure and excessive use in CD and UC was carried out using marginal logistic regression. The univariate analyses made it possible to select (*p* < 0.1) the exploratory variables to be evaluated in the multivariate models (selection of variables using a backward stepwise method with a threshold of 0.05 for remaining within the model) in each of the two diseases.

The statistical analyses were carried out in SAS (SAS institute, Cary, NC, USA) version 9.4.

## 3. Results

In total, 534 patients were included in France at four university hospitals, experts in the management of IBD: 324 patients with CD, 205 with UC, and 5 with unclassified colitis. The results for these five latter patients are not presented below due to their limited number.

### 3.1. Patients’ Characteristics and Treatments

At the last patients’ assessment, disease activity was moderate or severe in a minority of patients (CD: 25.0%; UC: 19%). For both IBDs, it was routinely assessed using patient reported outcomes (stool frequency and abdominal pain for CD and stool frequency and rectal bleeding for UC) and the IBD-Disk for disease-related burden [18].

Overall, 76.3% of patients with CD and 68.5% with UC have received previous treatment with at least one immunosuppressant or biotherapy/Janus kinase (JAK) inhibitor for their IBD; 93.5% and 98.0%, respectively, were on treatment at the time of inclusion in the study. Table 1 shows the patients’ previous and current treatments. They had already received or were currently treated with 5-aminosalicylic acid (5-ASA) (CD: 48.0%, UC: 92.7%), thiopurines (75.6%, 69.2%), other immunosuppressants (26.9%, 30.1%), TNF inhibitors (93.2%, 78.5%), anti-integrins (23.5%, 52.7%), interleukin (IL)-12/23 inhibitors (32.0%, 12.7%), and/or JAK inhibitors (1.3%, 11.8%). The current use of a biological therapy (TNF inhibitor, anti-integrin, IL-12/23 inhibitor) or JAK inhibitor was more frequently observed in the cases of a previous prescription of immunosuppressants (CD: 69.1% vs. 50.0%, UC: 66.1% vs. 26.7%) and, more specifically, thiopurines (CD: 59.9% vs. 50.0%, UC: 60.0% vs. 26.7%).

### 3.2. Corticosteroid Exposure and Excessive Use within the Last 12 Months

**Figure 1:**
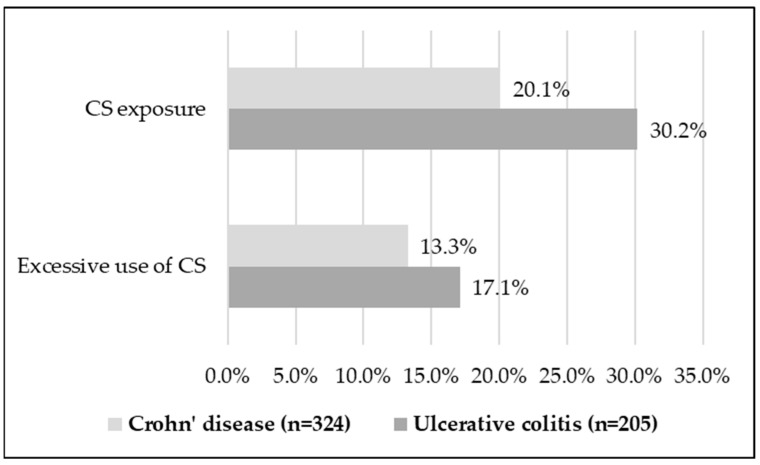
Corticosteroid (CS) use and excessive use within the last 12 months.

In the past 12 months, oral CS exposure was 20.1% (95% CI [16.1–24.8]) in CD patients and 30.2% [24.4–36.8] in UC patients. Excessive use was observed in 13.3% [10.0–17.4] of patients for CD and 17.1% [12.5–22.8] of patients for UC (Figure 1). CS exposure and excessive use were reported less frequently among patients with CD than for patients with UC (OR: 0.56, 95% CI [0.42–0.74], *p* < 0.0001 and 0.69 [0.54–0.89], *p* = 0.0042, respectively). When using CS, osteoprotective medication was only prescribed for a minority of patients, irrespective of the type of IBD studied (CD: 23.1%, UC: 19.4%).

Table 2 details the factors associated with CS exposure and excessive use within the last 12 months. Hence, disease activity assessed at the last patient visit was the independent factor most strongly associated (*p* < 0.01) with a risk of CS exposure and excessive use in CD (OR: 3.41 and 3.44) and in UC (OR: 7.29 and 6.90). Furthermore, the likelihood of CS exposure or excessive use increased with the number of previous biotherapies in patients with CD (OR: 1.66 and 1.71, *p* < 0.001). In patients with UC, the current use of 5-ASA was associated with CS exposure or excessive use (respective OR: 1.64 and 2.98; *p* < 0.01).

## 4. Discussion

The results of the DICE study highlighted the frequent use of CSs within the last 12 months in patients with IBD, particularly in UC (30.2% vs. 20.1% in CD, *p* < 0.0001) in the four participating French centers. Among those, CS excessive use, as defined by the British Society of Gastroenterology [13], represented more than half of cases for each of the two diseases (17.1% in UC and 13.3% in CD). These results are consistent with those observed in a study on British standard medical practice conducted in 2016 with CS exposure and excessive use in 30.0% and 14.9% of 1176 study patients with CD or UC, respectively [19]. This British study also showed a significantly greater incidence of CS use in UC patients compared to CD patients but only in those with moderately to severely active disease (77.2% vs. 57.0%, *p* = 0.003) as well as excessive CS use (42.6% vs. 28.1%; *p* = 0.026). By comparison, our data showed that moderate-to-severe UC and CD, as assessed by physicians at the last assessment, were significantly associated with the use and excessive use of CSs (*p* = 0.0002 and 0.0007, respectively).

Although the proportions of IBD patients with CS use or excess use we observed in France remain high, they are nonetheless lower than those reported for other chronic inflammatory diseases in adults, such as rheumatoid arthritis, for which approximately half of patients receive CSs [20]. In addition, CS exposure in IBD in France was lower than those observed in the international DICE study [CS: 32.7% of 1133 UC and 24.0% of 2618 CD; CS excessive use: 24.1% and 17.3%] [21], or in Germany alone [CS: 43.2% and 29.1%; CS excessive use: 20.9% in total] [22]. In the UK, 24.1% of the patients with CD had an excess use of CSs (vs. 13.3% in France) as well as 17.3% of patients with UC (vs. 13.3% in France) [23]. These differences relative to the French data can be explained by the expertise of the four participating French centers and their broad access to the various treatments available or under development. 

In the four participating French centers involved, as for the entire DICE study, CS exposure and excessive use were more frequently observed in patients with UC than in those with CD. This difference may be explained by the fact that marketing authorization was granted later for the anti-TNFs in UC than in CD. The same assumption was proposed by Selinger et al. to explain the higher rates of CS use and excess they reported in moderate/severe UC patients included in the 2016 British study [19]. While current 5-ASA use was associated with a likelihood of receiving CSs (excessively or not) in UC patients, it was the number of previous biological treatments that was significantly associated with these practices in patients with CD. In both diseases, however, disease activity deemed moderate or severe (versus mild or inactive) during the patient’s last assessment was an explanatory factor in both for the use of CSs and their excessive use.

The DICE study has several limitations. Due to its primary objective (i.e., to assess the oral CS exposure and excessive use in IBD patients), the study questionnaire was voluntarily short to favor the exhaustiveness of the cases to be reported during the last 12 months. As a result, in France, the four participating centers each completed a mean number of 133.5 online questionnaires for their IBD patients, and this leads us to believe that we approached actual prevalence rates in these French centers. However, this methodological choice led us to not ask physicians, for example, to fully describe the disease history of each included patient or the excessive use of CSs according to the four criteria by which it is defined [13]. Consequently, such data were not taken into account in the research of potential factors associated with CS exposure and excessive use in CD and UC. In addition, the French participating university centers were highly experienced in the management of patients with IBD and could not be considered as representative of the whole French population of hospital-based gastroenterologists.

## 5. Conclusions

The DICE study provided a snapshot of CS exposure in IBD in French university hospitals. The implementation of subsequent similar studies would enable us to monitor changes in these practices over time and to seek the lowest possible dose and the shortest duration of treatment for an optimal benefit/risk ratio, as has already been achieved in other inflammatory chronic diseases [24,25,26]. Furthermore, the routine monitoring of CS exposure in centers involved in the management of patients with IBD could also raise clinicians’ awareness of these prescriptions and of the risk of CS excessive use in routine medical practice.

## Figures and Tables

**Table 1:** Previous and current treatments for patients with inflammatory bowel disease.

Treatments	Crohn’s Disease (n = 324)			Ulcerative Colitis (n = 205)		
	Previous Treatment	Current Treatment	Treatment Not Taken	Previous Treatment	Current Treatment	Treatment Not Taken
5-ASA	110 (43.0%)	13 (5.1%)	133 (52.0%)	119 (61.7%)	60 (31.1%)	14 (7.3%)
Thiopurine	180 (58.6%)	52 (16.9%)	75 (24.4%)	107 (54.9%)	28 (14.4%)	60 (30.8%)
Other immunosuppressants	70 (22.7%)	13 (4.2%)	226 (73.1%)	51 (26.4%)	7 (3.6%)	135 (69.9%)
TNF inhibitor	118 (36.5%)	183 (56.7%)	22 (6.8%)	79 (38.5%)	82 (40.0%)	44 (21.5%)
Anti-integrin	29 (9.0%)	47 (14.6%)	247 (76.5%)	30 (14.6%)	78 (38.0%)	97 (47.3%)
IL-12/23 inhibitor	50 (15.5%)	53 (16.5%)	219 (68.0%)	17 (8.3%)	9 (4.4%)	178 (87.3%)
JAK inhibitor	4 (1.3%)	0 (0.0%)	316 (98.8%)	18 (8.8%)	6 (2.9%)	180 (88.2%)

5-ASA, 5-aminosalicylic acid; IL, interleukin; JAK, Janus kinase; TNF, tumor necrosis factor. Number of missing data (Crohn’s disease, ulcerative colitis): 5-ASA (68, 12), thiopurine (17, 10), other immunosuppressants (15, 12), TNF inhibitor (1, 0), anti-integrin (0, 0), IL-12/23 inhibitor (2, 1), JAK inhibitor (4, 1).

**Table 2:** Factors associated with CS use and excessive use within the last 12 months.

	Crohn’s Disease Odds Ratio [95% CI] (*p*-Value)		Ulcerative Colitis Odds Ratio [95% CI] (*p*-Value)	
	Univariate Analyses	Multivariate Analyses ^§^	Univariate Analyses	Multivariate Analyses ^§^
**Corticosteroid exposure within the last 12 months**				
Disease activity classed as moderate or severe vs. mild or inactive at the last assessment	**3.72 [1.75–7.91] (0.0007)**	**3.41 [1.58–7.35] (0.0018)**	**5.76 [2.72–12.21] (<0.0001)**	**7.29 [2.60–20.47] (0.0002)**
Number of previous biological therapies * (for 1 treatment)	**1.77 [1.29–2.41] (0.0003)**	**1.66 [1.18–2.32] (0.0032)**	**1.36 [1.11–1.67] (0.0030)**	
Ongoing immunosuppressants	0.89 [0.52–1.53] (0.6823)		**1.64 [0.97–2.78] (0.0666)**	**2.44 [1.24–4.80] (0.0098)**
Ongoing biological therapy * or JAK inhibitor	**0.34 [0.18–0.64] (0.0008)**		**0.57 [0.37–0.88] (0.0104)**	**0.58 [0.38–0.90] (0.0144)**
Ongoing 5-ASA ^#^	/		**1.75 [1.01–3.03] (0.0473)**	**1.64 [1.14–2.38] (0.0084)**
**Corticosteroid excessive use within the last 12 months**				
Disease activity classed as moderate or severe vs. mild or inactive at the last assessment	**3.84 [1.93–7.62] (0.0001)**	**3.44 [1.68–7.04] (0.0007)**	**6.37 [2.69–15.10] (<0.0001)**	**6.90 [2.90–16.43] (<0.0001)**
Number of previous biological therapies * or JAK inhibitors (for 1 treatment)	**1.84 [1.48–2.27] (<0.0001)**	**1.71 [1.38–2.12] (<0.0001)**	**1.48 [1.14–1.92] (0.0029)**	
Ongoing immunosuppressants	0.99 [0.41–2.42] (0.9889)		1.03 [0.38–2.78] (0.9485)	
Ongoing biological therapy * or JAK inhibitor	**0.42 [0.24–0.72] (0.0019)**		**0.57 [0.31–1.03] (0.0629)**	
Ongoing 5-ASA ^#^	/		**3.33 [2.21–5.01] (<0.0001)**	**2.98 [1.72–5.15] (<0.0001)**

5-ASA, 5-aminosalicylic acid; CI, confidence interval; JAK, Janus kinase. * *Tumor necrosis factor* inhibitor, anti-integrin, or interleukin-12/23 inhibitor. ^#^ Ongoing 5-ASA: parameter exclusively studied for ulcerative colitis (given the limited number of patients with Crohn’s disease in this case), ^§^ Selection of variables using a backward stepwise method (*p* < 0.05). Variables presented in bold font were included in the multivariate models (*p* < 0.1). Data from 322 patients with Crohn’s disease were used for the two multivariate models. For ulcerative colitis, data from 180 patients were used for analysis of corticosteroid exposure and data from 193 patients were used for excessive use.

## Data Availability

The data supporting the conclusions of this article will be made available by the authors upon reasonable request.
